# Intestinal Microbial Ecology and Environmental Factors Affecting Necrotizing Enterocolitis

**DOI:** 10.1371/journal.pone.0083304

**Published:** 2013-12-30

**Authors:** Roberto Murgas Torrazza, Maria Ukhanova, Xiaoyu Wang, Renu Sharma, Mark Lawrence Hudak, Josef Neu, Volker Mai

**Affiliations:** 1 Department of Pediatrics, College of Medicine University of Florida, Gainesville, Florida, United States of America; 2 Department of Epidemiology, College of Public Health and Health Professions and College of Medicine and Emerging Pathogens Institute, University of Florida, Gainesville, Florida, United States of America; 3 Department of Pediatrics University of Florida College of Medicine, Jacksonville, Florida, United States of America; Indian Institute of Science, India

## Abstract

Necrotizing enterocolitis (NEC) is the most devastating intestinal disease affecting preterm infants. In addition to being associated with short term mortality and morbidity, survivors are left with significant long term sequelae. The cost of caring for these infants is high. Epidemiologic evidence suggests that use of antibiotics and type of feeding may cause an intestinal dysbiosis important in the pathogenesis of NEC, but the contribution of specific infectious agents is poorly understood. Fecal samples from preterm infants ≤32 weeks gestation were analyzed using 16S rRNA based methods at 2, 1, and 0 weeks, prior to diagnosis of NEC in 18 NEC cases and 35 controls. Environmental factors such as antibiotic usage, feeding type (human milk versus formula) and location of neonatal intensive care unit (NICU) were also evaluated. Microbiota composition differed between the three neonatal units where we observed differences in antibiotic usage. In NEC cases we observed a higher proportion of Proteobacteria (61%) two weeks and of Actinobacteria (3%) 1 week before diagnosis of NEC compared to controls (19% and 0.4%, respectively) and lower numbers of Bifidobacteria counts and Bacteroidetes proportions in the weeks before NEC diagnosis. In the first fecal samples obtained during week one of life we detected a novel signature sequence, distinct from but matching closest to *Klebsiella pneumoniae*, that was strongly associated with NEC development later in life. Infants who develop NEC exhibit a different pattern of microbial colonization compared to controls. Antibiotic usage correlated with these differences and combined with type of feeding likely plays a critical role in the development of NEC.

## Introduction

Necrotizing Enterocolitis (NEC) is the most devastating intestinal disease in neonates.[1] On the basis of large, multicenter, neonatal network databases from the United States and Canada, the mean prevalence of the disorder is about 7% among infants with a birth weight between 500 and 1500 g but can vary markedly among centers.[2] The most recent data from different Neonatal Intensive Care Units in the National Institutes of Child Health Neonatal Network show a range of 4 to 19 percent in infants less than 28 weeks gestational age[3] and the range from Vermont Oxford Neonatal Intensive Care Units from 2010[4] shows a range from 2.2% to 8.3% (1^st^ and 3^rd^ quartiles) in babies born less than 1500 grams. These inter-unit variances suggest that differences in practice or other variables may contribute to the pathogenesis of this devastating disease.

An integral link between microbial dysbiosis, an exaggerated inflammatory response and NEC was hypothesized over a decade ago.[5] Maintenance of intestinal integrity and promotion of postnatal intestinal growth would accordingly require a delicate balance between intestinal microbiota and the immune system of premature infants, which can be affected by various environmental factors. Aberrant microbial colonization patterns or abnormal responses to normal microbiota might disrupt this balance and contribute to the development of NEC.

Studies using culture-based techniques have demonstrated differences in the intestinal microbiota of patients who subsequently developed NEC versus controls.[6] More recent studies using molecular methods to evaluate fecal microbiota from unaffected preterm infants, as well as some infants in whom NEC developed and from whom samples were obtained before and during NEC, suggest that this disease is associated with unusual intestinal microbes. [7]–[10] Although various microbes have been cultured from blood and stools in outbreaks of NEC at single institutions,[6], [11], [12] no single organism has consistently been implicated across centers.[13] The human microbiome project,[14] in conjunction with technologic advances that allow for the molecular identification of a vast array of microbes that are difficult or impossible to culture from the intestine, has given us new tools for generating evidence to test the “abnormal colonization hypothesis”.[15] Numerous environmental factors may contribute to intestinal microbial colonization patterns that predispose to NEC. Epidemiologic studies show a correlation between length of prior antibiotic use and the occurrence of NEC. [16], [17] The use of human milk versus formula, a factor known to be protective against NEC [18], also is likely to relate to altered microbial colonization.

In our previous study using high throughput sequencing, we demonstrated a bloom of Proteobacteria and several differences in operational taxanomic units (OTUs) prior to the onset of NEC.[9] Furthermore, we detected several operational taxonomic units (OTUs) associated with NEC status that did not have exact matches in Genbank, although they matched closest to *Klebsiella* in the class γ-proteobacteria.

There is little information currently available about variances and dynamics in microbiota associated with neonatal intensive care in different hospitals. Differences in the hospital environment and clinical practice such as routines in antibiotic administration and feeding can contribute to the establishment of distinct microbiota pattern in each unit. This has important implications for our ability to generalize findings regarding specific patterns of microbial dysbiosis in the pathogenesis of NEC in a specific NICU. In this current study, we address these issues by testing the hypotheses that a) the ontogeny of fecal microbiota differs in infants who subsequently develop NEC from those who remain free of NEC and b) environmental factors (e.g., antibiotic exposure, diet) may help explain the variance in NEC prevalence at different NICUs.

## Materials and Methods

### Ethics Statement

Written informed consent was obtained from the infants' parents and investigations were conducted according to the principles expressed in the Declaration of Helsinki. The study including consent procedure was approved by the UF Health Institutional Review Board 01.

### Study Design

Premature infants born at a postmenstrual age ≤32 weeks without major congenital anomalies or malformations were enrolled at three University of Florida affiliated hospitals shortly after birth. Two control infants were selected and matched to each NEC case infant by postmenstrual age, birth weight, hospital of birth, and date of birth (+/− 2 months). We could not match an appropriate second control infant for one of the cases, resulting in a total of 18 case and 35 control infants. NEC cases included only those infants with definite clinical and radiologic signs (pneumatosis intestinalis and/or portal venous gas) or necrotic bowel at surgery.

Weekly stool samples from study infants were collected from diapers beginning with the first available stool (meconium) and continuing until discharge, for immediate storage at −80°C. The samples analyzed from cases included those collected 2 weeks before NEC (15±3; days prior to NEC), 1 week before NEC (8.4±2.6 days prior), and the sample closest to diagnosis of (2.5±2 days prior) NEC. Samples from matched control infants were chosen during the same week of life at which the samples from the cases were obtained. For 12 out of the 18 infants that later developed NEC, and matched controls, one of the samples collected before NEC diagnosis represented the very first stool sample that was obtained during week one of life. These samples were analyzed in a subanalysis.

Written informed consent was obtained from the infants' parents and investigations were conducted according to the principles expressed in the Declaration of Helsinki. The study including consent procedure was approved by the Institutional Review Boards of all three hospitals.

### Microbiota Analysis

#### DNA extraction and quality control by denaturing gradient gel electrophoresis (DGGE)

DNA was extracted from 200–300 mg fecal samples using a modified Qiagen stool DNA extraction protocol that included a bead beating step.[19] We used DGGE analysis of the V6–V8 region as described previously for initial quality control.[7]

#### 16S rRNA sequence analysis

DNA was amplified using a primer set based on universal primers 27F (AGAGTTTGATCCTGGCTCA) and 533R (TTACCGCGGCTGCTGGCAC) to which titanium adaptor sequences and barcodes were added. Cleansed PCR products were pooled in equimolar amounts and submitted for sequencing using 454-Titanium chemistry. From the resulting raw data set, low quality sequences or sequences with a length less than 150 nucleotides were removed. We used the ESPRIT-tree algorithm, which maintains the binning accuracy of ESPRIT[20] while improving computational efficiency to bin sequences into Operational Taxonomic Units (OTUs) using similarity levels from 99% (species/strain level) to 80% (phylum level). We used QIIME[21] to calculate Chao rarefaction diversity and UniFrac distances [22] for comparing α and β diversity respectively.

For comparison between the current and the earlier sequence dataset we pooled sequences from both reads and reassigned OTUs using ESPRIT-tree. We then identified OTUs that contained the sequences that were found to differ between cases and controls in either dataset to determine sequence distribution in them.

#### qPCR based quantification of Bifidobacteria

We used a *Bifidobacteria* specific primer set (F: 5′ TCG CGT C(C/T)G GTG TGA AAG 3′; R: 5′ CCA CAT CCA GC(A/G) TCC AC 3′, annealing temperature 58°C) to quantify the amounts of *Bifidobacteria* genome equivalents in fecal samples. Duplicate vials containing 10 ng of DNA were included in each reaction and DNA purified from *B. longum* was used to generate the standard curve. Samples with less than one genome equivalent/ng of DNA were considered as negative.

### Statistics

Paired Student's *t*-test was used for normally distributed data. A chi squared- test and Fisher's exact were used to evaluate demographic data and clinical characteristics as appropriate. Two-tailed *p*-values were calculated and *p*<0.05 was considered to be statistically significant. To test for a difference in the abundance of OTUs a paired chi square test was followed by Fisher exact test. We adjusted for an expected high false discovery rate by increasing the requirement for statistical significance to *p*<0.01.The QIIME package was used to calculate *p*-values for differences in UniFrac distances.

## Results

### Patient characteristics and clinical outcomes

Baseline characteristics are summarized in table 1. The mean postmenstrual age in case and control infants was 28±2.36 weeks. Mean birth weight was 1187±371 grams for both groups combined, with an almost 20% less mean weight in NEC cases that did not reach statistical significance. The incidence of NEC was 12.4% for Gainesville and 6.8% for Jacksonville during this study period. There were no significant differences in clinical characteristics and major co-morbidities between the two groups (Table 2).

**Table 1:** Baseline Characteristics of the Infants. (Mean ± SD).

Characteristic	NEC (N = 18)	Control (N = 35)
**Birth weight – g**	1073±394	1246±350
**Gestational age at birth – wk**	27.4±2.6	28.5±2.2
**Male sex – no./total no. (%)**	12/18 (66.7)	17/35 (48.6)
**Type of Milk – no./total no. (%)**		
**Breast Milk**	5/18 (27.8)	20/35 (57.1)
**Formula**	3/18 (16.7)	3/35 (8.6)
**Both**	10/18 (55.6)	12/35 (34.2)
**Mode of delivery – no./total no. (%)**		
**Vaginal**	9/18 (50)	12/35 (34.3)
**C-section**	9/18 (50)	23/35 (65.7)
**Use of antenatal corticosteroids—no./total no. (%)**		
**Any**	3/18 (16.7)	10/35 (28.6)
**Full course**	11/18 (61)	20/35 (57)
**Prenatal antibiotic exposure**	13/18 (72.2)	29/35 (82.9)
**Apgar score at 1 min**	5±2.8	5.3±2.7
**Apgar score at 5 min**	7±2.3	7.6±1.7
**Positive pressure ventilation (bag and mask)**	11/18 (61.1)	16/35 (47)
**Continuous positive airway pressure (CPAP)**	6/18 (33.3)	13/35 (37.1)
**Intubation and mechanical ventilation**	10/18 (55.6)	14/35 (41.2)
**Day of life of Development of NEC**	17.83±12.8	
**Total days on antibiotics prior to NEC or sample**	6.2±6.9	4.9±3.4

**Table 2:** Major Clinical Outcomes. # cases/# in group (%).

	NEC (N = 18)	Control (N = 35)	P value
**Bronchopulmonary dysplasia**	5/18 (27.8)	5/35 (14.3)	0.23
**Intraventricular hemorrhage**	4/18 (22.2)	4/35 (11.4)	0.35
**Patent ductus arteriosus**	6/18 (33.3)	7/35 (20)	0.26
**Periventricular leukomalacia**	1/18 (5.6)	3/35 (8.6)	0.12
**Retinopathy of prematurity**	2/18 (11.1)	8/35 (22.9)	0.27

### Type of feeding

Of the infants in the control group 57.1% received exclusively maternal milk compared to 27.8% in cases (p<0.05).

### Antibiotic Exposure

As shown in table 1, there was similar prenatal exposure to antibiotics administered to mothers. Postnatally, the duration of antibiotic exposure prior to NEC did not differ between cases and controls (6.2±6.9 days versus 4.9±3.4 days, mean ± S.D.). We did not detect significant differences in the individual antibiotics prescribed before diagnosis in NEC cases compared with controls (Table 3). While we observed some differences in antibiotic administration between the NICU in Gainesville and the two NICUs in Jacksonville (Figures S1 and S2), with a length of antibiotic therapy of 22 days in Jacksonville and 7 days in Gainesville at the 75^th^ quartile, none of these differences reached statistical significance. Prior to the development of NEC among cases born in Gainesville 73% were exposed to Ampicillin and Gentamicin and 18% to Oxacillin, which was not used in Jacksonville, whereas among NEC cases born in Jacksonville, 100% were exposed to Ampicillin and Gentamicin and to other antibiotics not used in Gainesville such as Vancomycin (40%), Piperacillin/Tazobactam (28%), Azithromycin (14%), Cefazolin (14%), Clindamycin (14%),.

**Table 3:** Antibiotic exposure before NEC # prescribed antibiotic/# in group (%).

	*NEC*	*Control*
**Ampicillin**	15/18 (83.3)	31/35 (88.6)
**Azithromycin**	1/18 (5.6)	4/35 (11.4)
**Cefazolin**	1/18 (5.6)	0/35
**Cefotaxime**	0/18 (0)	4/35 (11.4)
**Ceftazidime**	0/18 (0)	1/35 (2.9)
**Clindamycin**	1/18 (5.6)	0/35 (0)
**Fluconazole**	0/18 (0)	1/35 (2.9)
**Gentamicin**	15/18 (83.3)	30/35 (85.7)
**Oxacillin**	2/18 (11.1)	1/35 (2.9)
**Piperacillin/tazobactam**	2/18 (11.1)	2/35 (5.7)
**Vancomycin**	3/18 (16.7)	2/35 (5.7)

### Microbiota analysis

**Figure 1:**
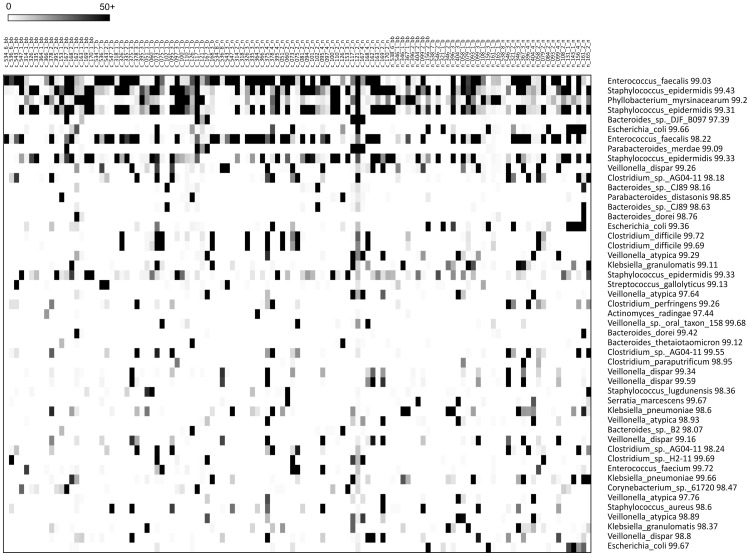
Dominant OTU's. Each row represents a separate OTU with the closest match in the database listed on the right. Each column represents an individual sample from both cases and controls at each of the three time points. OTUs are listed in order of dominance with the most dominant on the top. Darker color indicates higher number of sequences in this OTU in an individual sample (see color code in left corner). Darkest shade indicates 50+ sequence reads obtain for that OTU in that sample.

The distribution of dominant OTUs from all three NICUs is shown in Figure 1. The most dominant OTU's detected in both cases and controls matched closest to Enterococcus sp., Staphylococcus sp, Phyllobacterium sp., Bacteroides sp., Escherichia sp., Parabacteroides sp., and Veillonella sp.

**Figure 2:**
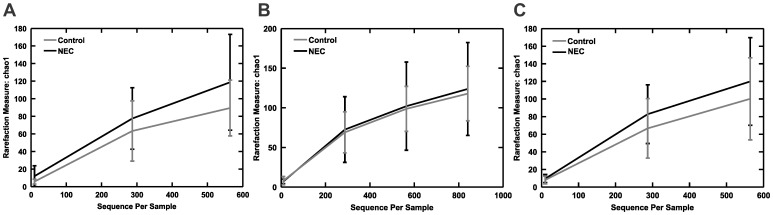
Chao rarefaction diversity. Chao diversity was calculated from the OTU distribution A) 2 weeks before diagnosis of NEC; B) 1 week before diagnosis of NEC; and C) Week of diagnosis of NEC. As a measure of beta (within sample) diversity it is an estimate of the expected total number of OTUs detected in the sample if sequenced to completion.

Species richness, a measure of alpha (within sample) diversity as determined by a Chao1-based estimate of the total numbers of OTUs present, did not differ between cases and controls at any of the three time-points (Figure 2). This indicates that the total numbers of different bacterial species present in the gut was stable during the observation period.

**Figure 3:**
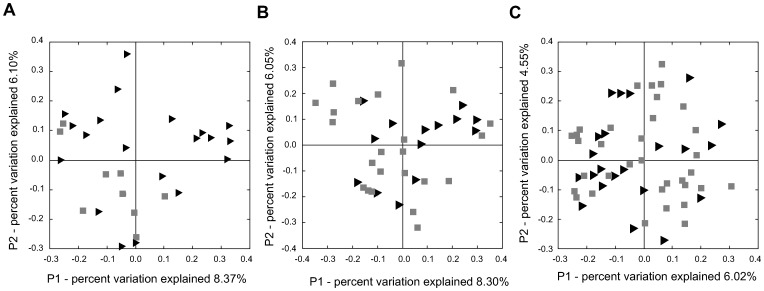
Unifrac diversity measures. Principal component analysis (PCA) of overall diversity based on UniFrac (unweighted) metric A) 2 weeks before; B) 1 week before; and C) week of diagnosis of NEC. Squares represent controls and triangles represent cases. P1 is component 1 and P2 component 2.

In contrast, using the UNIFRAC metric of beta (between samples) diversity, we detected a difference in overall microbiota composition between cases and controls two weeks before diagnosis (Figure 3). At this time point, samples from cases clustered together more closely compared to samples from controls (p<0.05). This suggests that although the total number of bacteria present did not change over time or differ by case status, the kinds of bacteria present and their proportions did differ. However, no clustering by case status was detected during the following weeks.

**Figure 4:**
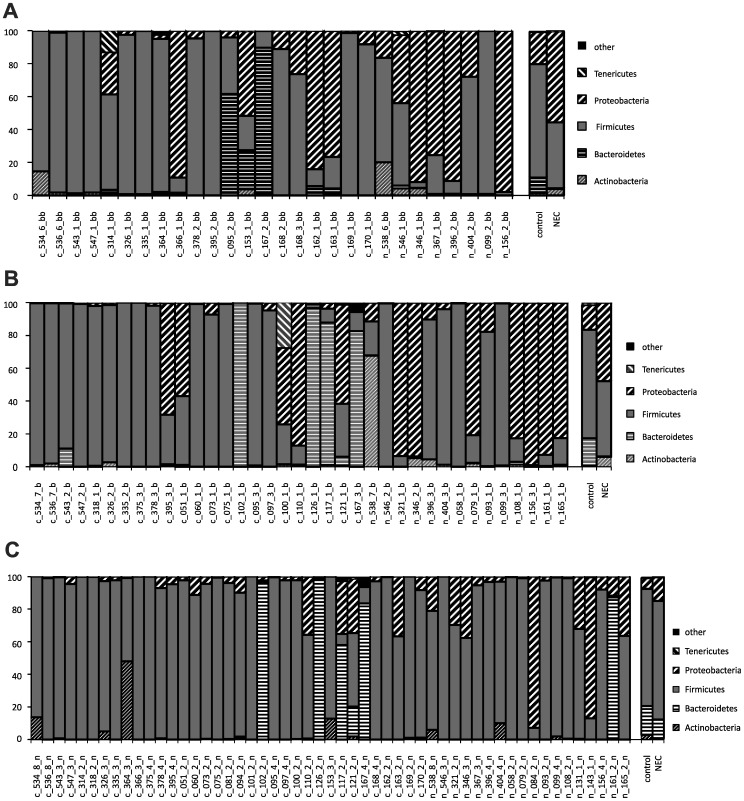
Changes in proportions of bacterial phyla. Proportions of the major bacterial phyla at A) two weeks before, B) one week before, and C) during week of NEC diagnosis for individual samples from controls (c_###) and NEC cases (n_###) and means for samples combined by NEC status (control, NEC).

**Figure 5:**
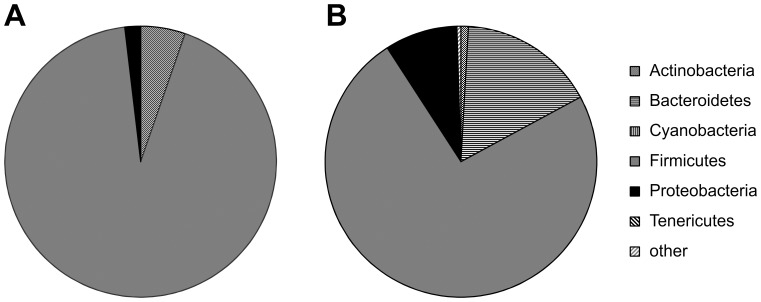
Differences in the proportions of prevalent bacterial phyla, based on 454 16S rRNA sequencing, in controls at week 0 in A) Jacksonville and B) Gainesville.

The clustering by case status two weeks before diagnosis can be attributed largely to an increased proportion in *Proteobacteria* and a decreased proportion of *Bacteroidetes* in cases, which is most evident when the mean for each group is considered (Figure 4). A matched analysis comparing the distribution of the major phyla showed no difference at week of diagnosis in any of the major phyla. Proteobacteria decreased with advancing age in both cases and controls. At 2 weeks before diagnosis proteobacteria were increased in cases compared to controls, P<0.001. At one week before diagnosis Actinobacteria and Proteobacteria was increased in cases compared to controls, P<0.001. When we examined the abundances of phyla by center we found no difference in cases but detected in controls a clear difference by center at all time points. During week of diagnosis (in matched cases) we observed in controls from Gainesville a higher proportion of Bacteroidetes, which were almost completely absent in controls from Jacksonville (P<0.01), and Proteobacteria at the cost of Firmicutes (Figure 5).

**Figure 6:**
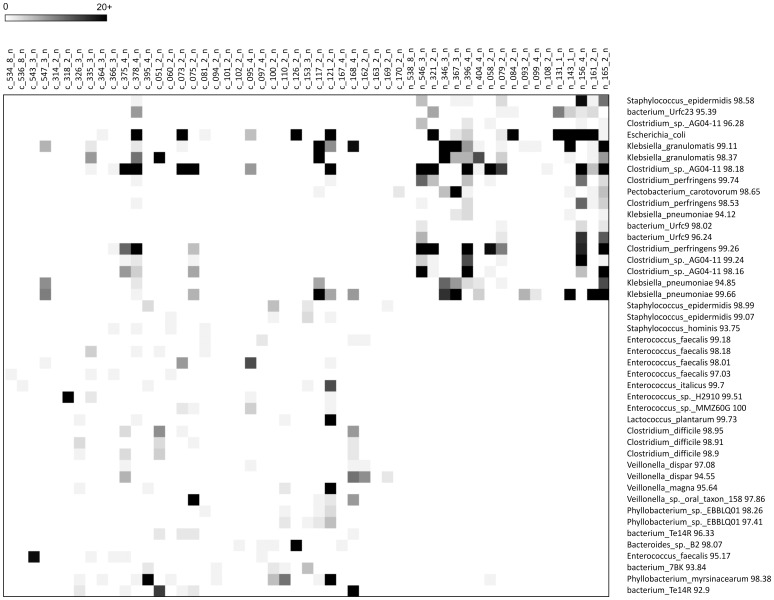
Heat map of selected OTU (98% similarity) correlating with NEC status during the week of diagnosis. Controls are shown on the left (c_###) and NEC cases on the right (n_###). The number of sequences detected per sample for each OTU is indicated by degree of shading, with the darkest shade correlating with the highest number of sequences. OTUs more frequently observed in NEC cases are shown on the top and OTUs less frequently observed are shown on the bottom.

In the analysis of the distribution of all OTUs between cases and controls at each time point we detected various OTUs, matching to different bacterial phyla/families/classes, that significantly differed in prevalence/frequency by case status. During the week of diagnosis we observed such OTUs in all but one of the cases (Figure 6). These OTUs might represent either novel pathogens contributing to NEC or commensals that can thrive under the conditions in the gut when NEC develops. Multiple OTUs matching closest to the potentially pathogenic *Klebsiella granulomatis*, *Klebsiella pneumoniae* and Clostridium perfringens were detected close to the time of diagnosis more frequently in cases. Other significantly increased OTUs in NEC cases matched closest to *Staphylococcus epidermidis*. Many OTUs frequently observed in controls at early time points were completely missing in cases, suggesting that bacteria normally colonizing the gut of control infants didn't do so in cases. Other OTUs were only detected in cases, some of them in significant numbers especially at the two time points closest to diagnosis.

**Figure 7:**
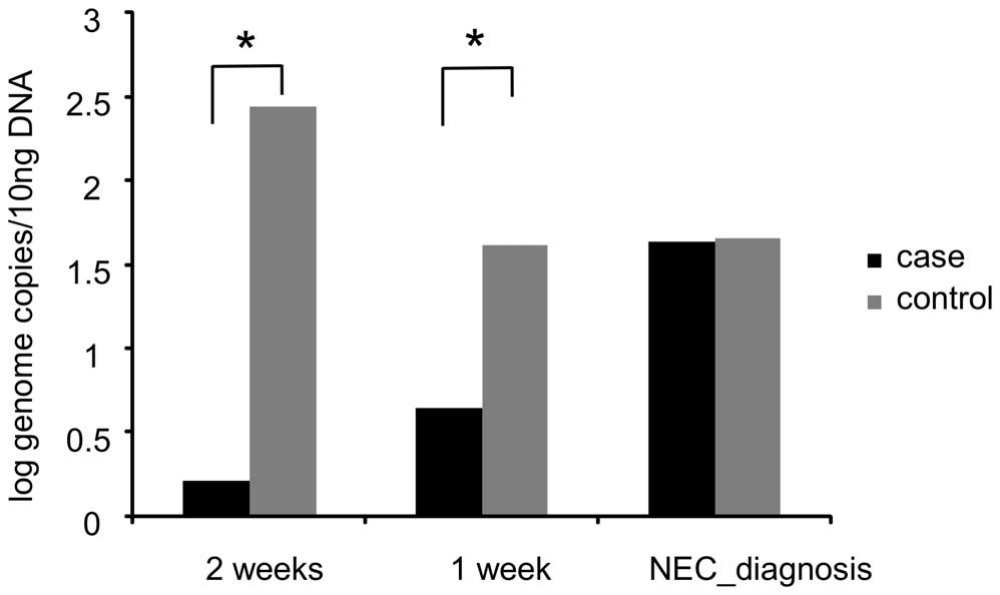
qPCR for fecal counts of Bifidobacteria. * p-value 2 weeks before NEC <0.05; **p-value week of NEC <0.05.

We used a bifidobacteria targeting qPCR approach to quantify colonization in cases and controls. This was necessary because the ‘universal’ primer set used in our 16S rRNA sequence analysis is biased against bifidobacteria, a group of bacteria known to frequently colonize the infant gut. While numbers of bifidobacteria appeared lower in cases than in controls during the weeks before diagnosis (p<0.05) there was no difference during the week of diagnosis (Figure 7).

We then performed a sub-analysis in all 12 of the NEC cases, and 23 matched controls, for which we had sequence data available for the very first stools collected during week one of life. In these samples we observed a particularly strong association between NEC risk and an OTU distinct from but matching closest to K.p.. This OTU was detected in 11/12 infants that later developed NEC, compared to only 9/23 matched controls (p<0.01). Furthermore, in 5/12 NEC cases this OTU represented more than 10% (range 10-63%) of all 16S rRNA sequences that were obtained, compared to only 1/23 controls in which this OTU was >10% (Figure S3).

## Discussion

A symbiotic relationship between intestinal microbiota and the host is established soon after birth. Preterm infants have developmental delays and encounter environmental factors that differ from term infants and challenge the development of a normal symbiosis. Mode of delivery, feeding type (breast versus formula), and antibiotic use may predispose the infant to the development of various diseases including NEC.[23] This prospective study shows that various aspects of intestinal microbiota composition differ in infants who develop NEC compared to controls. Two weeks before diagnosis, there is an increased proportion in *Proteobacteria* and a decreased proportion of *Bacteroidetes* in cases. At one week before diagnosis *Actinobacteria* and *Proteobacteria* were increased in cases compared to controls. *Proteobacteria* decreased with advancing age in both cases and controls. The increased mean proportion of *Actinobacteria* in cases is an observation that differs from our earlier report, but it is mostly due to a single case in whom *Actinobacteria* contributed the majority of all sequences (Figure 4B). While in cases, the proportion of *Firmicutes* (containing clostridia, lactobacilli and gram positive cocci) consistently increased from 2 wk (34%) to 1 wk (52%) prior to onset of NEC reaching its peak (72%) during the week of NEC there was less of an increase in controls. Many OTUs frequently observed in controls at early time points were lacking in cases, suggesting that bacteria conferring a benefit did not colonize in a timely manner. Other OTUs were significantly increased in cases, suggesting a potentially pathogenic role. Such OTUs include those matching closest to the potentially pathogenic *Klebsiella granulomatis*, *Klebsiella pneumoniae* and *Clostridium perfringens*, but also one that matched *Staphylococcus* epidermidis, a known skin commensal that has previously been associated with NEC risk.[24] Previous studies[11] including our own[9] reported similar observations. The strongest association that we detected was for a K.p.-like OTU that was significantly enriched in the very first stool samples obtained after birth from a subset of infants that later developed NEC. The consistent association in our and other studies with Klebsiella[6], [9] and especially OTUs closest to but clearly distinct from K.p. suggests that we might have identified a novel K.p. -like pathogen.

Our data are to some extent consistent with previous studies[6], [9] that suggested that the microbiota, particularly amounts of *Proteobacteria*, differ in infants who subsequently develop NEC compared to those who do not.[10] While we did not detect a difference in overall richness (chao-1 alpha diversity) as seen at the time of development of NEC in the Wang study[8], we did detect microbiota clustering, based on UNIFRAC metric, in cases two weeks before diagnosis. Previously, we suggested a *Proteobacteria* bloom associated with NEC onset.[9] In the current study, with a greater number of subjects, such a bloom was not evident, but instead, with increasing age *Proteobacteria* remained high in cases but declined in controls. Differences in gestational age, birth weight and week of NEC onset, in this compared to the earlier report, likely contributed to this discrepancy. Nevertheless, in an earlier report[25] of microbial composition in inflamed intestinal tissue surgically removed from ileum of 24 infants with NEC found that 49% consisted of Proteobacteria, compared to other phyla comprised of: 30.4% were Firmicutes, 17.1% were Actinobacteria and 3.6% Bacteroides. The relative proportion of these phyla is consistent with those seen in the feces of the infants who subsequently developed NEC in both our previous study[9] and the current one. With respect to the longitudinal timeline we believe that the paradox that *Proteobacteria* proportion declined and that the *Firmicutes* increased as the week of NEC approached in the NEC group further impresses the significance that the characteristics of microbiome during the earlier postnatal period drives the susceptibility of premature infants to NEC.

The differences in microbiota compositions observed in controls between the Gainesville and Jacksonville study sites are likely multifactorial, but differences in the administration of individual antibiotics could be a factor contributing to the site differences. However, numbers were too small to make definitive conclusions. Future multi-site studies will need to consider geographic and temporal difference in antibiotic administration. These differences between NICU microbial compositions suggest that we may not be able to extrapolate all data from one NICU to all others.

Many factors can affect a normal pattern of intestinal colonization such as mode of delivery, type of feeding, and antibiotic exposure.[23], [26], [27] C-section versus vaginal were not related to development of the disease, but consumption of exclusive human milk versus commercial formula was higher in patients who did not develop NEC. Previous studies suggest that infants born via c-section and/or that are predominantly fed infant formula have a similar intestinal pattern of colonization composed predominately of *Proteobacteria* such *Escherichia coli*, and *Firmicutes* including some potential pathogens such as *Clostridia* and *Staphylococcus*.[26], [27] In contrast, those infants fed predominantly human milk develop a more desirable “healthy” intestinal flora composed of *mainly Lactobacilli*, *Bacteroides* and *Actinobacteria* (*Bifidobacterium*).[26], [28] The current study did not have sufficient number of subjects to compare specifically microbial composition in human milk versus formula fed infants who subsequently developed NEC.

The predominance of *Proteobacteria* one and two weeks before diagnosis of NEC and *Actinobacteria* one week before diagnosis of NEC in cases compared to control could in part suggest that the difference in type of feeding or exposure to antibiotics played a role. Indeed, antibiotic exposure may reduce the diversity of intestinal microbiota, delay the colonization of beneficial bacteria and potentially predispose preterm neonates to NEC.[23], [29] Antenatal exposure to antibiotics increased risk of NEC in a retrospective clinical study.[30] In our study antenatal exposure to antibiotics did not differ between the two groups (NEC versus control). We were able to detect a suggestive difference in days of treatment with antibiotics, albeit not statistically significant, when we compared centers. Previous studies [29], [31] have shown that duration of antibiotic exposure postnatally is associated with an increased risk of NEC among neonates without prior sepsis. During the study period the incidence of NEC was 12.4% in Gainesville and 6.8% in Jacksonville. As no significant difference in antibiotic use was observed among cases it is possible that the observed differences in antibiotic exposures among controls resulted in the selection for microbes in each NICU unit that correlated with NEC risk.

A recent study[10] found no *Propionibacteria* in 9 NEC infants compared to their presence in 56% of control infants. Our findings from 18 NEC infants appear to differ since the proportion of *Actinobacteria* (includes *Propionibacteria*) was greater in the group that later developed NEC in our study. Whether this was due to differences in timing of sample collection or inter-neonatal intensive care unit differences in microbial ecology remains speculative.

In our study the abundance of *Bifidobacteria* was lower in cases than in controls. This was determined by qPCR analysis as the universal primers used for 16S rRNA sequence analysis contain multiple mismatches with the conserved target region and consequently amplify bifidobacteria poorly. *Bifidobacteria* have been described as beneficial bacteria for intestinal development and function^31^ and their higher prevalence may have been related to a greater use of breast milk in control infants.[32].

In summary, it appears that the microbiota of babies who subsequently develop NEC is different from those who do not. The two distinct forms of intestinal dysbiosis prior to the onset of NEC in the two studies from our group suggests that an abnormal pattern of colonization with predominance of *Proteobacteria* early in life or later in the days closer to the development of NEC may be associated with a greater risk for developing NEC. Differences in colonization patterns and NEC were seen depending on neonatal intensive care unit. The use of human milk versus formula also was associated with a lower NEC rate; this finding should be confirmed in other studies. Although the differences in antibiotic usage and human milk feeding could not be correlated to specific changes in microbiota, there were obvious differences prior to the development of NEC. These patterns of altered intestinal microbiota may be modulated by the different modalities of treatment that the infants have undergone. An improved understanding of the factors that establish a healthy intestinal microbiota or that induce dysbiosis offers opportunities for early interventions that reduce the risk of NEC. The consistent finding from now multiple studies that K.p.-like strains appear more frequently observed in NEC cases suggests that an hitherto unknown pathogen might contribute to the etiology in at least a subset of NEC cases.

## Supporting Information

Figure S1 **Antibiotic usage and exposure distributed by Center in NEC cases.**
(TIF)

Figure S2 **Antibiotic usage and exposure distributed by Center in controls.**
(TIF)

Figure S3 **Percentage of all sequence reads for the first fecal sample collected during week one of life from 12 NEC cases and 23 matched controls that matched to a OTU closest to but distinct from** ***Klebsiella pneumoniae*** **.**
(TIF)
