# Supplementary figures and images for: Intestinal Microbial Ecology and Environmental Factors Affecting Necrotizing Enterocolitis
Source: PLoS One. 2013 Dec 30;8(12):e83304. doi: 10.1371/journal.pone.0083304 (PMC3875440; doi:10.1371/journal.pone.0083304)

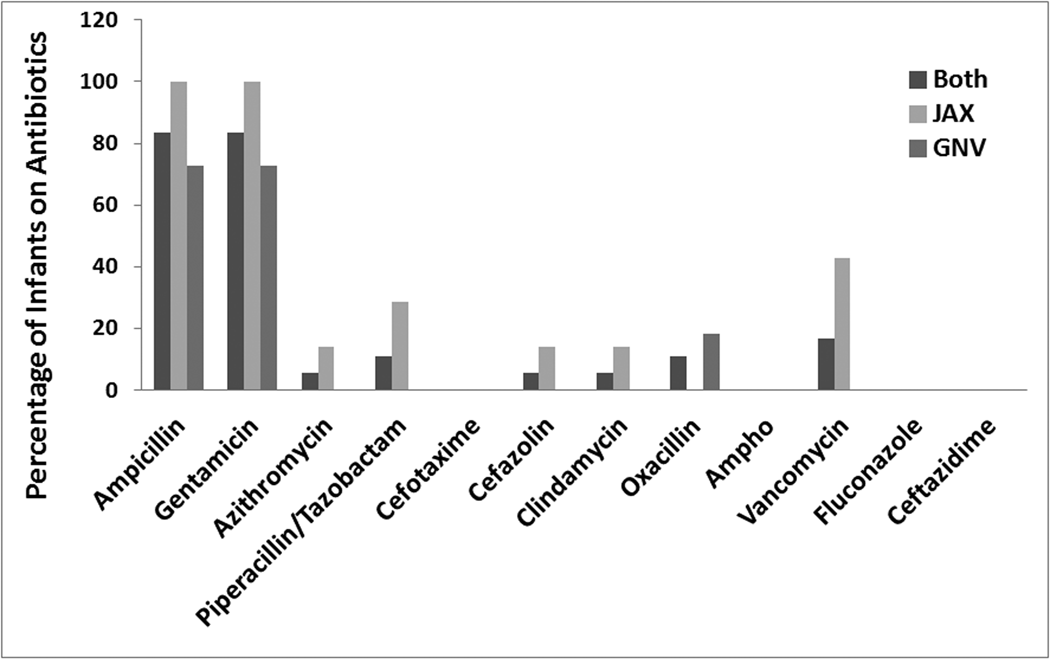

Supplement: Figure S1 — Antibiotic usage and exposure distributed by Center in NEC cases. (TIF) [file pone.0083304.s001.tif]

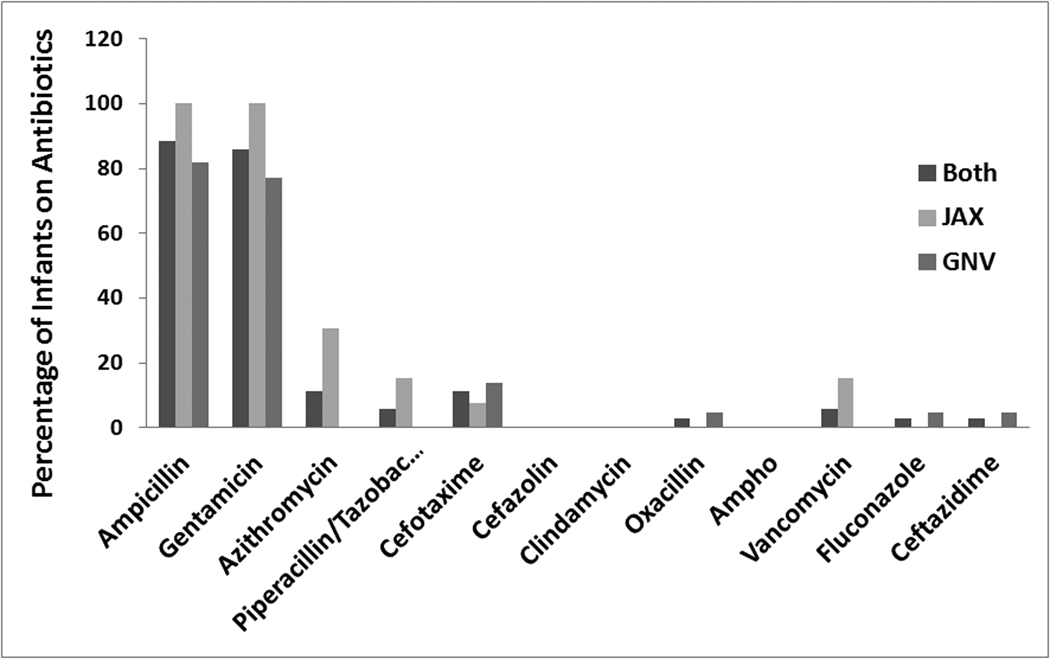

Supplement: Figure S2 — Antibiotic usage and exposure distributed by Center in controls. (TIF) [file pone.0083304.s002.tif]

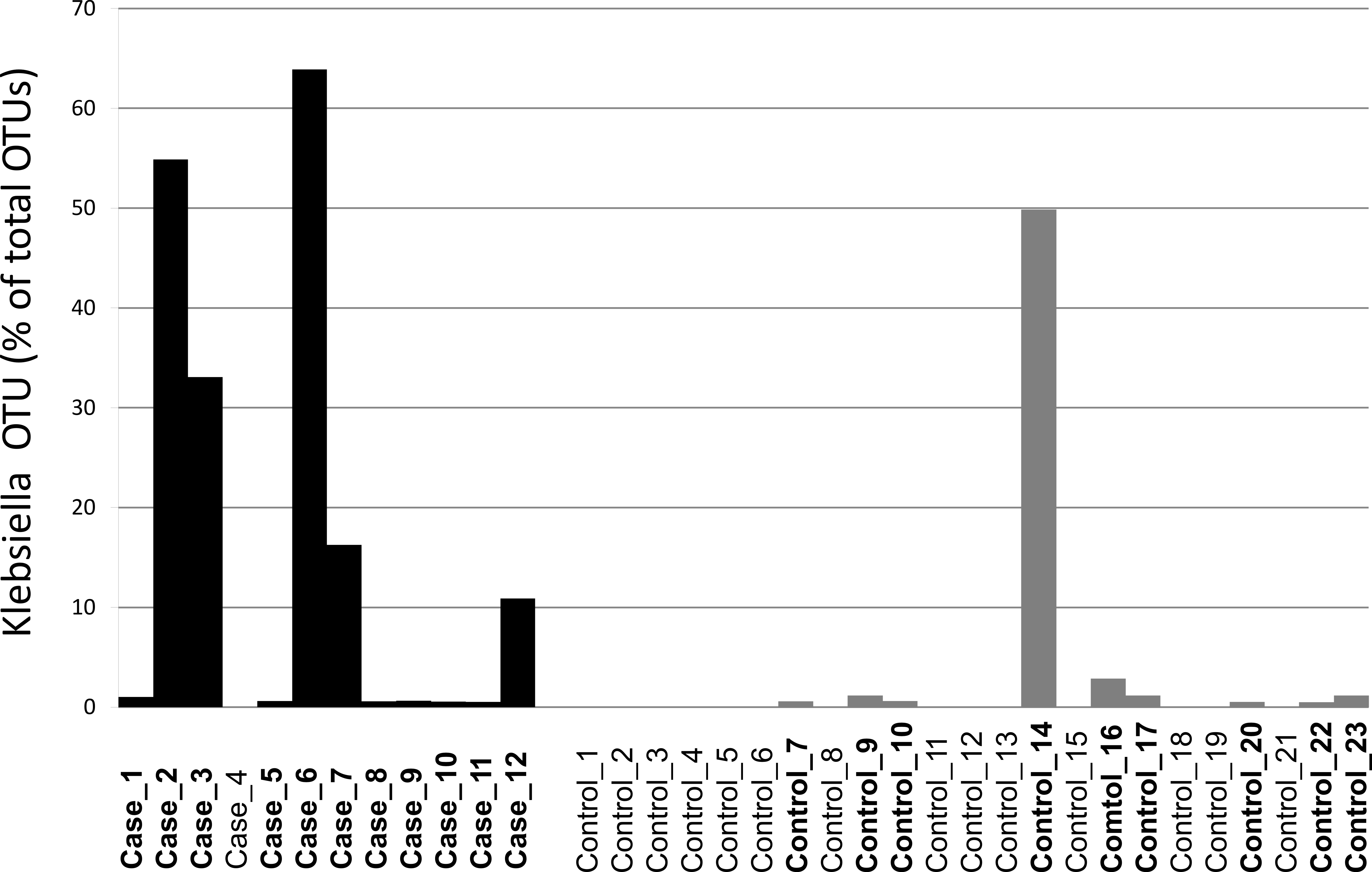

Supplement: Figure S3 — Percentage of all sequence reads for the first fecal sample collected during week one of life from 12 NEC cases and 23 matched controls that matched to a OTU closest to but distinct from Klebsiella pneumoniae . (TIF) [file pone.0083304.s003.tif]
